# Priming Effects of Focus in Mandarin Chinese

**DOI:** 10.3389/fpsyg.2019.01985

**Published:** 2019-08-30

**Authors:** Mengzhu Yan, Sasha Calhoun

**Affiliations:** School of Linguistics and Applied Language Studies, Victoria University of Wellington, Wellington, New Zealand

**Keywords:** alternatives, contrast, focus, prosody, syntax, Mandarin Chinese

## Abstract

Psycholinguistic research has long established that focus-marked words have a processing advantage over other words in an utterance, e.g., they are recognized more quickly and remembered better. More recently, studies have shown that listeners infer contextual alternatives to a focused word in a spoken utterance, when marked with a contrastive accent, even when the alternatives are not explicitly mentioned in the discourse. This has been shown by strengthened priming of contextual alternatives to the word, but not other non-contrastive semantic associates, when it is contrastively accented, e.g., after hearing “The *customer* opened the window," *salesman* is strongly primed, but not *product*. This is consistent with Rooth's (1992) theory that focus-marking signals the presence of alternatives to the focus. However, almost all of the research carried out in this area has been on Germanic languages. Further, most of this work has looked only at one kind of focus-marking, by contrastive accenting (prosody). This paper reports on a cross-modal lexical priming study in Mandarin Chinese, looking at whether focus-marking heightens activation, i.e., priming, of words and their alternatives. Two kinds of focus-marking were investigated: prosodic and syntactic. Prosodic prominence is an important means of focus-marking in Chinese, however, it is realized through pitch range expansion, rather than accenting. The results showed that focused words, as well as their alternatives, were primed when the subject prime word carried contrastive prosodic prominence. Syntactic focus-marking, however, did not enhance priming of focused words or their alternatives. Non-contrastive semantic associates were not primed with either kind of focus-marking. These results extend previous findings on focus and alternative priming for the first time to Chinese. They also suggest that the processing advantages of focus, including priming alternatives, are particularly related to prosodic prominence, at least in Chinese and Germanic languages. This research sheds light on what linguistic mechanisms listeners use to identify important information, generate alternatives, and understand implicature necessary for successful communication.

## 1. Introduction

The process of successful comprehension in spoken discourse involves more than understanding the words that are said. Listeners need to attend most carefully to the part of an utterance which gives the most important information, that which updates the common ground. Further, as the theme of this research topic attests, they frequently need to infer information which is not directly expressed in the utterance they are listening to, such as alternatives to one of the elements in the utterance. Focus-marking, e.g., by contrastive accenting, allows listeners to do this. For example, when a speaker says “The *customer* closed the window” (italics indicate a contrastive accent), this implies not simply that the customer closed the window, but also that *the customer* is the important information which updates an explicit or implicit ‘question-under-discussion’ (QUD) like “Who closed the window?” (Roberts, 1996), and that it is relevant that someone else, e.g., *the salesman*, could have closed the window. To make the communication successful, listeners must be able to successfully identify the focus, and thus infer the alternatives intended by the speaker even when these are not available in the context. Psycholinguistic studies since the 1970s have shown that focused words are indeed attended to more than defocused or unfocused words: they are recognized faster and remembered better (e.g., Cutler and Fodor, 1979; Birch and Garnsey, 1995; Cutler et al., 1997; Birch et al., 2000; Akker and Cutler, 2003) More recently, there has been mounting evidence that listeners activate alternatives in sentences like these, even when the alternatives are not explicitly mentioned in the discourse; and this activation is facilitated by contrastive accenting (e.g., Braun and Tagliapietra, 2010; Gotzner et al., 2016; Husband and Ferreira, 2016).

Focus-marking therefore has at least two key functions: to indicate the information which updates the common ground, and, following the alternative semantics theory proposed by Rooth (1992), to indicate contextually-relevant alternatives. There are a number of different linguistic means to indicate focus, including contrastive accenting (or prominence), certain syntactic constructions, e.g., clefts, and morphological markers (Féry and Ishihara, 2016). However, most of the psycholinguistic work in this area has concentrated on contrastive accenting. While some work has shown that clefting strengthens attention and memory for focused words (Birch and Garnsey, 1995; Birch et al., 2000; Kember et al., 2016a,b), to our knowledge, no previous studies have investigated whether other focus-marking mechanisms also activate alternatives, e.g., clefting, in the absence of prosodic focus-marking. Across languages, morphosyntactic means of marking focus are as common as prosodic, and if focus is the underlying mechanism this should be the case. However, if the activation of alternatives is rather related to prosodic prominence, which enhances the salience of the prominent word, and therefore its processing, these other focus-marking mechanisms would not activate alternatives.

Further, to our knowledge, all of the studies in this area have been carried out on Germanic languages, which have very similar prosodic systems. In this paper, we report on a cross-modal lexical priming study carried out in Mandarin Chinese (hereafter Chinese). Prosodic prominence is a key marker of focus in Chinese, however, prominence in Chinese is marked differently from Germanic languages, through pitch register expansion rather than pitch accenting (Xu, 1999). Focus can also be marked by cleft constructions in Chinese (Fang, 1995; Paul and Whitman, 2008). The study therefore expands the cross-linguistic validity of the effects. The study looks at priming of subject arguments in spoken sentences; looking at whether subject words and their alternatives are primed by syntactic as well as prosodic cues to focus in Chinese.

In section 2, we will define focus, drawing on the theoretical literature, and present the prosodic and syntactic markers of focus in Chinese explored in this study. We then review previous studies regarding the effect of focus on speech processing, including the recent research on the role of focus in priming alternatives.

## 2. Focus and Focus-Marking

Focus is a key part of information structure. During a discourse, speakers build a *common ground* of propositions relevant to the context they believe to be established with the other speaker(s) (Stalnaker, 1974; Clark, 1996). To facilitate this, each utterance has an *information structure*, i.e., each argument, predicate, etc. is marked as to how it refers back to, alters and/or updates the common ground (Chafe, 1976; Féry and Krifka, 2008; Krifka, 2008). One key kind of marking is *focus-marking*. There are two main definitions of focus, which are in principle orthogonal to each other (Calhoun, 2010; Vallduví, 2016). Under the first the focus, or rheme, is the part of the utterance which updates the common ground, or is new in relation to the current question-under-discussion (QUD) (see e.g., Ginzburg, 1994; Roberts, 1996; Vallduví, 2016). We will call this QUD-focus. Under the second, the focus, or contrast, indicates “the presence of alternatives that are relevant for the interpretation of linguistic expressions" (Rooth, 1992; Krifka, 2008, p. 247). We will call this contrastive focus. Both of these are illustrated in the following (bold indicates the prosodic prominence, F shows the focus):^1^


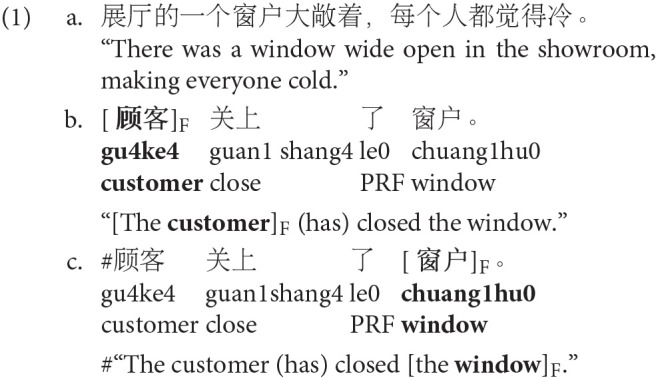


In (1b), 顾客 “the customer” is the QUD-focus as it updates the common ground, giving new information on 窗户 “the window,” which was mentioned in (1a), i.e., there is an implied question of 谁关上了窗户*?* “Who closed the window?” The focus is marked by prosodic prominence on 顾客 “customer.” This prominence can also indicate focus according to the second definition, i.e., contrastive focus, implying a contextually-appropriate set of alternatives to 顾客 “customer,” e.g., 店员 “salesman.” Non-contrastive associates, i.e., words that are semantically associated with 顾客 “customer,” but cannot replace it in the sentence, e.g., 产品 “product,” are not in the alternative set. Likewise, alternatives to the unfocused argument, i.e., 窗户 “window,” are not implied. As can be seen in this example, while QUD-focus and contrastive focus are in principle separable, in practice the same constituent in a sentence is often focused by either definition. When the sentence is said with prosodic prominence on 窗户 “window,” as in (1c), this is incongruent, as focus on the object does not match the context by either focus definition: an implied question of 顾客关上了什么*?* “What did the customer close?” is odd as 顾客 “the customer” was not mentioned; likewise, alternatives to 窗户 “window” are odd as only the window is mentioned as being open.

**Figure 1:**
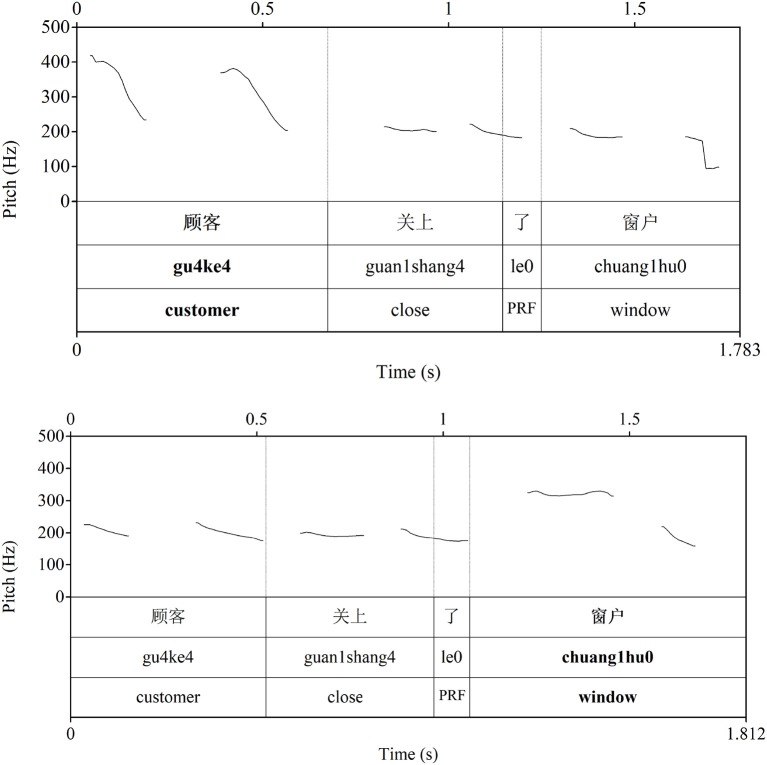
A comparison of primary prosodic prominence on the subject **(Top)** and object **(Bottom)** in Chinese (see text for details).

In Chinese, prosodic prominence is a key marker of focus (Xu, 1999; Wang and Xu, 2006; Chen and Gussenhoven, 2008). Prosodic prominence is not realized by pitch accenting, as in Germanic languages, as the lexical tone determines the local F0 curve of each syllable. Rather, prosodic prominence is realized through pitch register. The pitch range in the focused word is expanded, and the region following the focus compressed (e.g., Xu, 1999; Wang and Xu, 2006; Chen and Gussenhoven, 2008) (see Figure 1). The focused word is also realized with longer duration and higher mean intensity (e.g., Xu, 1999; Chen and Gussenhoven, 2008; Chen et al., 2009). When the focus is on the subject, the following pitch range is heavily reduced (as in Germanic languages). When the focus is on the final object, which is the default position for primary prominence in Chinese, the pitch range in the pre-focal region is still relatively wide (again, similar to Germanic languages).

As mentioned above, prosodic prominence is not the only means of marking focus. Across languages, there are a number of other cues that mark focus including morphosyntactic cues (e.g., clefts) and focus particles (e.g., *only, even*) (Féry and Ishihara, 2016). For instance, in Chinese, like in many languages, clefts can mark focus (Lambrecht, 2001; Paul and Whitman, 2008), as in the following:


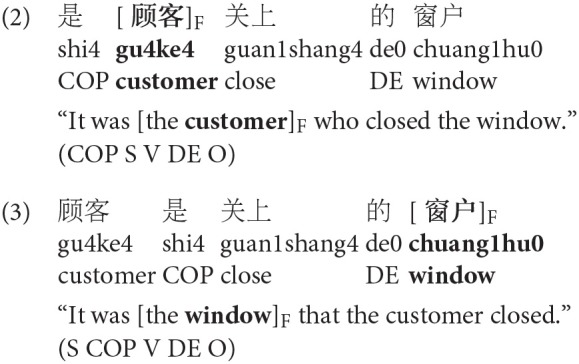


Clefts are marked morphosyntactically in Chinese using the 是…的 (*SHI…DE*) construction, without changing the word order (Fang, 1995). For instance, for subject focus, as in (2), the copula 是 (*SHI*) occurs immediately before the subject, and 的 (*DE*) either before or after the object. When 的 (*DE*) appears before the object, the sentence is past tense (Hole, 2011). In this paper, we use the pre-object 的 (*DE*) (see Simpson and Wu, 2002; Paul and Whitman, 2008; Hole, 2011 for an overview of the SHI…DE cleft construction). For object focus, as in (3), the copula 是 (*SHI*) occurs before the verb, and the 是 …的 (*SHI…DE*) construction does not change the word order. (2) marks focus on the subject, and like (1b) would be compatible with the context in (1a), while (3) marks object focus and would not be coherent following (1a).

**Figure 2:**
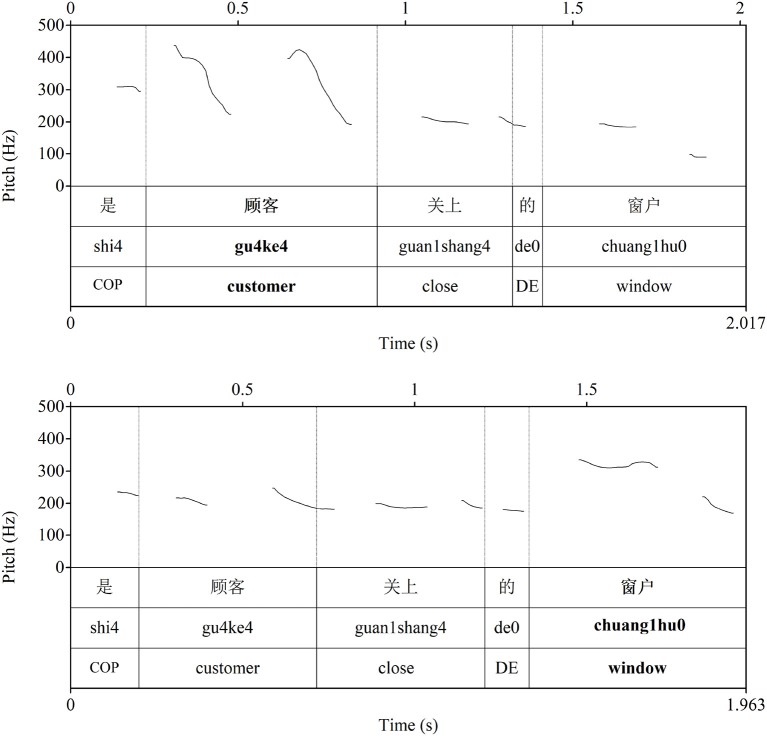
Examples of the prosody of an ScleftS **(Top)** and ScleftO **(Bottom)** sentence in Chinese.

The prosodic prominence normally falls on the cleft head, as shown in this example (see the top example in Figure 2). In (2), the cleft marks both QUD-focus on the cleft head, and contrastive focus, implying alternatives to it (Fang, 1995; É Kiss, 1998; Lambrecht, 2001). Further, it has been claimed that clefts have an exhaustive implication that focus-marking with prosodic prominence does not necessarily have (É Kiss, 1998; Krifka, 2008). The cleft rules out other alternatives in the context of that proposition. For example, it would be possible after (1b) to continue 而且店员帮助了她 “and the salesman helped her,” but this would be not possible, or pragmatically odd, after (2).

While the prosodic prominence normally falls on the cleft head, it can also fall in the main clause (see the bottom example in Figure 2). In these cases, the QUD-focus is usually analyzed as being in the main clause, i.e., cued by the prosodic prominence (Prince, 1978; Delin and Oberlander, 1995; Lambrecht, 2001; Hole, 2011; Hedberg, 2013; Feldhausen and Vanrell, 2015), as in the following (note in both Chinese and English it is possible to have a secondary prominence, or accent, on the cleft head, however, the nuclear prominence is in the main clause):


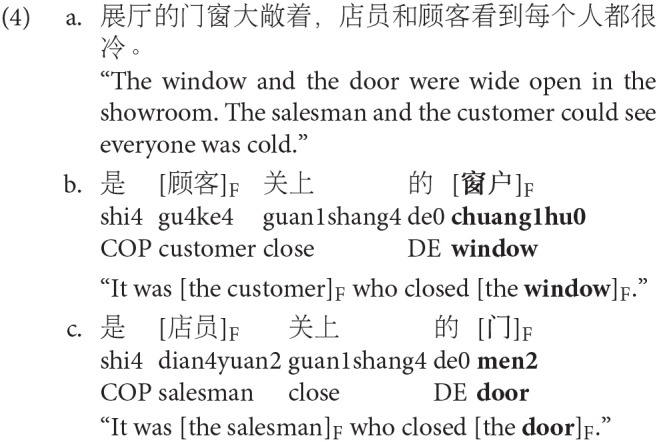


Following the analysis of Büring (2003) (see also Constant, 2014; Riester, 2018), based on (4b) and (4c), (4a) sets up an implied question 谁关上了什么*?* “Who closed what?,” which can be divided into two sub-questions 顾客 关上 了什么 *?* “What did the customer close?' and 店员 关上 了什么 *?* “What did the salesman close?,” which is answered by the second focus in each of the following sentences. This part updates the common ground. However, importantly for our purposes, alternatives are implied for both the subject and object, as shown by the multiple focus-marking. As well as the alternatives to what was closed (窗户, 门 “window, door”), there are alternatives to the subject in the implied question, or contrastive topics (顾客，店员 “customer, salesman”). That is, the syntactic and prosodic cues mark contrastive focus on different words (the subject and object respectively). While this kind of construction, a cleft with prosodic prominence in the main clause, has received little attention in the experimental literature, it is well attested in corpus-based studies of naturally occurring speech in English (Prince, 1978; Delin and Oberlander, 1995; Lambrecht, 2001; Hedberg, 2013), and it is shown to be used in certain contexts in natural speech in Chinese (Hole, 2011).

## 3. The Effects of Focus on Language Processing

In this section, we review the literature showing the effects of focus on the processing of focus-marked words. Almost all of this work has been on English and other Germanic languages, so most studies discussed are necessarily on these languages. We start by briefly reviewing studies looking at the processing of focus in general, and then review research on the role of focus-marking in word activation in lexical decision tasks, the method employed in this study.

It has long been established that focused words enjoy a processing advantage over unfocused or defocused words. These earlier studies assume a QUD-focus definition of focus. In phoneme-monitoring experiments, phonemes in focused words or the words whose preceding intonation contour predicts a focus are recognized faster (Cutler, 1976; Cutler and Fodor, 1979; Akker and Cutler, 2003; Ip and Cutler, 2017). Focused words are also remembered better (Birch and Garnsey, 1995; Birch et al., 2000; Kember et al., 2016a). For example, when a word in an *it*-cleft was presented later in a memory task, participants were faster in confirming that they had previously seen the word than when it was not in focus (Singer, 1976; Birch and Garnsey, 1995; Birch et al., 2000). These experiments used written stimuli, so the primary cue to focus was syntactic. However, it has been shown that readers generate “implicit prosody” while reading (Fodor, 1998, 2002; Stolterfoht et al., 2007; Jun, 2010; Jun and Bishop, 2015). Here, it is most likely the implicit prosody would have the nuclear accent in the cleft head (see above). Recently, Kember et al. (2016a) used a similar memory task to look at the effect of focus in spoken sentences in Korean. They found that both prosodic and syntactic cues to focus enhanced memory for focused words, with syntactic and syntactic+prosodic cues most effective.

More recently, another line of studies, using eye-tracking, have given the first psycholinguistic evidence of focus as facilitating activation of alternatives, i.e., for the contrastive focus definition of focus. These studies showed that contrastive accenting biases listeners to look at contrastive referents that are available in their visual display, compared to non-contrastive accenting which shows no bias (Dahan et al., 2002; Weber et al., 2006; Ito and Speer, 2008; Watson et al., 2008; Dennison, 2010; Kurumada et al., 2014). To our knowledge, though, the only two types of focus-marker investigated in these studies are contrastive accenting and focus particles (Kim, 2012; Kim et al., 2015). Another line of work has looked at whether different types of focus-marking facilitate memory for foci and their alternatives in discourse contexts (Fraundorf et al., 2010, 2013; Spalek et al., 2014). Fraundorf et al. (2010, 2013) found contrastive accents and font emphasis respectively facilitate memory for foci and correct rejection of alternatives, while Spalek et al. (2014) found focus particles facilitate memory for mentioned alternatives, but not focused words themselves, on accented words in discourse contexts.

Most importantly for our purposes, there have been studies looking at the role of focus-marking in word activation. Since the 1970s (Swinney et al., 1979), the activation of words given different linguistic primes has been investigated using cross-modal lexical decision tasks. These studies have shown that single words prime themselves (identity prime) and their semantic associates; but while identity priming is consistent in sentence contexts, semantic associative priming is not (Norris et al., 2006). For example, Norris et al. (2006) showed that when participants heard an auditory prime *seat*, they were quicker to recognize an identical printed target *seat* was a word (compared to an unrelated control target *river*); likewise they were quicker to respond to a semantically associated target *chair*. However, when the prime word was in a sentence, e.g., *He gave up the seat for me out of some form of courtesy*, participants were still faster to respond to the identical target *seat*, but not the semantic associate *chair*. Norris et al. (2006) then tested a number of variables that could affect semantic associative priming in sentence contexts. They found that this priming was only significant when the sentence was truncated immediately after the prime word, or when there was a contrastive accent in the sentence, whether or not this was on the prime word. They speculated that the latter result may be because the accent caused the listeners to attend to the sentence as a whole more carefully. They also suggest that the contextual relevance of the target to the meaning of the prime in the sentence may affect priming, although they do not directly link this to focus.

Braun and Tagliapietra (2010) provided a key insight into a possible reason for these results: whether the word is contrastively focused. According to alternative semantics theory, contrastive focus-marking should imply alternatives to the focused word. Hence, in a lexical decision task, alternatives should be activated when the prime in a spoken sentence is contrastively accented, but not when the prime is not. Braun and Tagliapietra (2010) compared semantic priming of the sentence-final object word in sentences with one of two intonation patterns in Dutch: contrastive, with contrastive accents on both the first and last content word in a sentence [e.g., (5a) and (5c)]; and neutral, with non-contrastive accents on these words [e.g., (5b) and (5d)]:

(5)
  a. In **Florida** he photographed a **flamingo**(**Contrastive - related prime**)
  b. In Florida he photographed a flamingo(**Neutral - related prime**)
  c. In **Florida** he photographed a **celebrity**(**Contrastive - control prime**)
  d. In Florida he photographed a celebrity(**Neutral - control prime**).

In their first experiment, after hearing the prime sentence, participants saw a target(e.g., *pelican*). The object in the prime sentence was either related to, and was a contextual alternative, to *pelican*, e.g., *flamingo* in (5a) and (5b); or it was unrelated, e.g., *celebrity* in (5c) and (5d). Participants were quicker to decide *pelican* was a real word after hearing the related prime *flamingo* compared to the unrelated control prime *celebrity* when the sentence-final object was contrastively accented (alternative priming). However, there was no time advantage when the sentence-final object (*flamingo* or *celebrity*) was not contrastively accented. Their second experiment examined the priming of non-contrastive associates (e.g., *pink*) that were not plausible replacements for *flamingo*. They found that non-contrastive associates were weakly primed regardless of the prosody. The priming of contrastive and non-contrastive associates was not directly compared in the two experiments. However, it seems fair to say that alternatives were primed more than non-contrastive associates when the prime was contrastively accented.

Husband and Ferreira (2016) also looked at semantic priming in sentences with either contrastive or neutral accents, finding a somewhat different pattern of results to Braun and Tagliapietra (2010). In their study, the prime word was a sentence-medial object or adjective, e.g.,:

(6)
  a. The museum thrilled the **sculptor** when they called about his work (**Contrastive**)
  b. The museum thrilled the sculptor when they called about his work (**Neutral**).

After hearing the sentence, participants saw a target which was either a contextual alternative (e.g., *painter*) or a non-contrastive associate (e.g., *statue*). Husband and Ferreira (2016) were interested in the time course of activation of the prime, or the stimulus onset asynchronies (SOA) from the prime word. In their first experiment, the SOA was 0 ms. This was similar to Braun and Tagliapietra (2010), however, as the prime word was non-final, this was while the sentence was still playing. Husband and Ferreira (2016) found all semantic associates were primed except for non-contrastive associates in the neutral prosody, as non-contrastive associates were less related to the semantic context and had less time to be activated. In their second experiment, the SOA was set at 750 ms. When the prime word was contrastively accented, the non-contrastive associates were responded to at the same speed as unrelated items while alternatives were faster, showing only the alternatives were primed. When the prime word had a neutral accent, both contrastive and non-contrastive associates were faster than the controls. Husband and Ferreira's (2016) explanation for the mechanism behind this was different to Braun and Tagliapietra (2010). They claim this shows all semantically related words are initially activated, but contrastive accenting prompts a selection mechanism whereby non-contrastive associates are rapidly deactivated, while contextual alternatives remain activated as they are likely to be relevant for interpretation. However, it should be noted that there were a number of other differences between the studies, including the details of how the contrastive/neutral accenting conditions were manipulated, and the time course of when the target was presented.

Braun and Tagliapietra (2010) and Husband and Ferreira (2016) only looked at priming of alternatives cued by contrastive accenting. A series of psycholinguistic experiments conducted by Gotzner and colleagues (Gotzner et al., 2016; Gotzner, 2017) explored the activation and processing of alternatives where the focus prime was marked by focus particles *only* and *even* in German. While contrastive accenting indicates the presence of relevant alternatives to the focus, focus particles add further semantic restrictions on the interpretation of those alternatives, e.g., *only* excludes the possible alternatives (similarly to the claimed effect of clefts discussed in section 2). Using both probe recognition tasks, and lexical decision tasks, they found focus particles slowed the recognition of mentioned alternatives, and the rejection of unmentioned alternatives. They attribute the result to an interference effect of the focus particle, due to increased competition between members of the alternative set.

Bringing together these studies, there is considerable evidence that focus, marked by contrastive accents, facilitates activation of alternatives to the focused word, in and out of a discourse context. However, there are a number of important gaps in our present knowledge of this process. Firstly, if focus-marking is the underlying mechanism, we should also expect identity priming to be strengthened for focus-marked words in sentence contexts, compared to non-focus-marked words. Focus-marked words should be more activated by either focus definition: they are part of the alternative set (Rooth, 1992), and they are important information as the QUD-focus (see the early findings on focus in phoneme-monitoring and memory tasks). While this has been shown in phoneme-monitoring and memory tasks (for contrastive accenting and clefting), it has not been looked in previous research using lexical decision tasks, to our knowledge. Secondly, the evidence is mixed as to whether focus affects priming in the absence of contrastive accenting. Syntactic focus-marking, i.e., clefting, was looked at in the earlier memory experiments (Birch and Garnsey, 1995; Birch et al., 2000), but not in the alternative priming studies (Braun and Tagliapietra, 2010; Husband and Ferreira, 2016). Focus particles, in addition to contrastive accenting, have been shown to slow processing in general, rather than further prime alternatives. Therefore, it is not yet established if other focus-marking mechanisms (e.g., clefts) facilitate priming of focused words and their alternatives in the absence of contrastive accents. If the underlying mechanism is focus-marking, this should be the case; however, it is not fully clear it is, and there are indications focus-marking apart from contrastive accenting can slow processing.

Thirdly, the priming effects of focus have only been looked at in a handful of closely related languages, i.e., English, Dutch, and German. It is therefore cross-linguistically important and interesting to see whether they can also be found in other language families, in this case, Mandarin Chinese. Very little research has been carried out in Mandarin Chinese on the processing advantage of focus. As discussed above, like English, in Chinese prosodic prominence is a primary marker of focus, although marked with phrasal prominence, rather than pitch accents. Therefore, we might expect these languages to be similar. In a phoneme-monitoring task in Chinese, Ip and Cutler (2017) showed that target phonemes in words were responded to faster when the preceding prosody predicted focus, in line with the findings in Germanic above (e.g., Cutler, 1976). More closely, our recent experiment (Yan et al., 2019) tested the role of contrastive prominence in priming focused words, contrastive alternatives and non-contrastive associates of subject nouns in canonical order sentences in Mandarin Chinese. The study followed a very similar design to the present one, except that the two sentence types compared were canonical word order sentences with contrastive prosodic prominence on the object (canonO in this study) or the subject (canonS, not included in this study). It was found that focused words and contrastive alternatives were recognized faster when the subject carried contrastive prominence (canonS) than when it did not (canonO). Non-contrastive associates were not primed in either of the conditions. However, we did not test the role of syntactic cues to focus.

In this paper, we report on a cross-modal lexical priming study testing the role of prosodic and syntactic focus-marking in facilitating priming of words and their alternatives in Mandarin Chinese. Note that for this study, focus-marking means contrastive focus: all of the focus-marking conditions compared have been shown to mark contrastive focus on the subject, but not necessarily QUD-focus (although some also mark QUD-focus), see section 2. The prime word was always the subject noun, with the target presented after the end of the sentence. We were interested in the priming effects after a longer course of processing, rather than immediate processing, as this is when effects of focus should be stronger (as per Husband and Ferreira, 2016). Braun and Tagliapietra (2010) and Husband and Ferreira (2016) looked at sentence-final (objects) or sentence-medial elements, so to our knowledge no studies in this area have yet tested subject nouns using cross-modal lexical priming paradigms. Subject nouns are interesting to look at, as previous work has shown that positional cues to focus affect processing ease (e.g., Repp and Drenhaus, 2015).

## 4. The Experiment

### 4.1. Research Questions

This experiment addressed the following research questions:

1. Is prosodic or syntactic F(ocus)-marking necessary for subject nouns to prime *themselves*? If not, do they strengthen the priming?
2. Is prosodic or syntactic F-marking necessary for subject nouns to prime their *contrastive alternatives*? If not, do they strengthen the priming?
3. Is prosodic or syntactic F-marking necessary for subject nouns to prime their *non-contrastive associates*? If not, do they strengthen the priming?

### 4.2. Methods

#### 4.2.1. Participants

Ninety-nine (79 females and 20 males) native Mandarin Chinese speakers (mean age = 20.77, SD = 1.92, age range = 18–26) were recruited from students at Henan Polytechnic University in China. 80 were from Henan province and 19 were from other Mandarin speaking provinces. They reported that they had received English education, but they did not speak other languages at home and were not fluent in any other languages. They had not lived outside China for more than 6 months. They received supermarket vouchers in recognition of their participation. None of them reported any hearing or reading difficulties.

#### 4.2.2. Materials and Design

Sixty critical sentences were constructed containing a prime word as the subject noun (see full list in the Supplementary Material). All sentences described a simple, plausible event in the past tense, using commonly occurring nouns and verbs. As much as possible, the event described by the verb and the object was not semantically related to the subject, so there were no potential semantic priming relationships within the sentence. Most of the sentences were subject-verb-object (SVO) sentences; six were subject-verb-preposition-object. They all had seven syllables in the canonical order version.

For each sentence, three sentence type versions were created, involving different focus-marking on the subject noun (see examples in Table 1): *no F-marking*, i.e., canonical word order with nuclear prominence on the object (canonO); *syntactic F-marking*, i.e., subject cleft with nuclear prominence on the object (ScleftO); and *prosodic+syntactic F-marking*, i.e., subject cleft with nuclear prominence on the subject. For each sentence, a quadruplet of four target types was constructed (identical item, contrastive alternative, non-contrastive associate, unrelated control). The contrastive alternatives could replace the subject nouns in the sentence. The non-contrastive associates were related to the subject nouns, but could not replace them in the sentence. The unrelated controls were not related to the subject nouns. All target words were not related to the objects and verbs to avoid being primed by them. Three sentence types and four target types resulted in twelve experimental conditions. 60 sentences were used to make 180 experimental sentences (60 sentences * 3 sentence types). Each sentence was paired with four target types, which gave a total of 720 experimental stimuli. Twelve lists of 60 experimental stimuli were constructed in a Latin square design. Each participant saw only one list.

**Table 1:** Sentence types, with F-marking, and target types used in the Chinese experiment (bold shows nuclear prominence; the information on F-marking refers only to the subject noun in each case).

**Sentence types**	**Examples**
canonO (no F-marking)	顾客关上了**窗户**
	“The customer closed the **window**.”
ScleftO (syntactic F-marking)	是顾客关上的**窗户**
	“It was the customer who closed the **window**.”
ScleftS (prosodic+syntactic F-marking)	是**顾客**关上的窗户
	“It was the **customer** who closed the window.”
**Target types**	**Examples**
Identical	顾客
	“customer”
Contrastive	店主
	“shop owner”
Non-contrastive	产品
	“product”
Control	陆地
	“land”

There were several further steps involved in preparing the experimental stimuli, which are described below. First, we describe a survey carried out to create semantic relatedness norms needed to control for semantic relatedness between target types. Second, for similar reasons, we controlled for word frequency between words across target types. Then we describe the recording and acoustic analyses of the experimental stimuli. Finally, we describe the construction of other items (fillers).

##### 4.2.2.1. Relatedness scores

The semantic relatedness between the non-identical targets and the subject nouns was tested, to be able to control for this in the analysis. Since there were no published association norms for Mandarin, the relatedness scores were collected from an online questionnaire constructed in Qualtrics (2017).

Seventy-five common disyllabic nouns were extracted from a Chinese word frequency corpus SUBTLEX-CH (Cai and Brysbaert, 2010). Seventy-five short sentences were constructed with the nouns as the subject. Then three other words were selected from the corpus for each sentence: contrastive alternative, non-contrastive associate and unrelated control, relative to the subject noun. These words were not related to, and could not replace, any other word in the sentence. Therefore, there were 75 quadruplets, each resulting in three pairs of ratings: subject noun vs. contrastive alternative, subject noun vs. non-contrastive associate, subject noun vs. unrelated control. Sixty-seven native Mandarin speakers from Henan Polytechnic University completed the online questionnaire. Each participant saw only one of the three pairs. They were asked to rate the relationship between two words from 1 “*not related at all*” to 7 “*highly related*” in the presence of a context sentence (e.g., *how related are “customer” and “salesman” in the sentence “The customer closed the window”*). Yan (2017) showed that context affects the relatedness scores. The participants who took part in the online questionnaire did not participate in the lexical decision task.

Following the survey, 60 sentences were chosen in order to have similar relatedness scores between the subject noun and both of the two types of associates, and also for the subject noun and the unrelated control to be as unrelated as possible. The mean relatedness score was 4.83 (SD = 1.88) for prime-contrastive (e.g., *customer-shop owner*), 5.05 (SD = 1.89) for prime-non-contrastive (e.g., *customer-product*), and 1.77 (SD = 1.34) for prime-unrelated (e.g., *customer-land*). Relatedness scores as the ordinal dependent variable and the relationship between two words in a pair as the independent variable were fitted into a cumulative link mixed model using the ordinal package in (R Core Team, 2017; Christensen, 2019). The results showed no significant differences between the prime-contrastive pair and the prime-non-contrastive pair (*z* = –1.586, *p* = 0.26). However, significant differences were found between the prime-control pair and the prime-contrastive pair (*z* = 21.17, *p* < 0.001) and between the prime-control pair and the prime-non-contrastive pair (*z* = 22.00, *p* < 0.001).

##### 4.2.2.2. Frequency

The log frequency of each target word was collected from SUBTLEX-CH (Cai and Brysbaert, 2010). The mean log frequency of the chosen items was 3.085 (SD = 0.44) for subject nouns, 2.916 (SD = 0.43) for contrastive alternatives, 2.790 (SD = 0.43) for non-contrastive associates and 2.917 (SD = 0.44) for unrelated controls. The log frequency of each word was fitted into an ANOVA, and the *post-hoc* Tukey test showed a significant difference between subject nouns and non-contrastive associates (e.g., *product-shop owner*) [*t*_(236)_=3.70, *p* = 0.002], though the frequencies between word types were controlled to be closely matched. No significant differences were found between the other groups (all *p*-values > 0.1).

##### 4.2.2.3. Recording and acoustic analysis

The sentences were recorded directly to hard drive using Praat (Boersma and Weenink, 2018) by a trained female native Mandarin speaker (first author) in a soundproof room at Victoria University of Wellington through a USB-based microphone (see Figure 1 above for examples of canonS and canonO, and Figure 2 for ScleftS and ScleftO). All sentences were checked impressionistically by two native Mandarin speakers for the location of prosodic prominence.

The acoustic measurements (duration, mean F0, max F0, min F0 and mean intensity) of words were obtained using ProsodyPro (Xu, 2013). As focus is marked through pitch range expansion in Chinese, F0 range was also calculated being the difference between max F0 and min F0. The measurements (duration, mean F0, F0 range, and mean intensity) were fitted as the dependent variable in separate linear mixed effects models, using the R package lme4 (Bates et al., 2015). The fixed effects initially included sentence type (canonO, ScleftO, ScleftS) and word position (subject, object) as well as the interaction between the two. Tone combination was also included, as tone affects syllable duration and F0 (e.g., Long, 1985). Word was the random effect. Each model was reduced to remove non-significant factors (see further section 4.2.4). The fitted values are provided in Table 2. The ANOVA tables of the final models for each measurement are provided in Table 3. Tone combination was a significant factor for duration, mean F0 and F0 range. All four models showed a significant interaction between sentence type and word position. In general, as Table 2 shows, in subject-stressed sentences, the subject was more prominent than the object, whereas in object-stressed sentences, the object was more prominent than the subject. Planned comparisons, which were run using the emmeans function in the lsmeans package (Lenth, 2016), showed that, within the same sentence type, prosodically focused subjects or objects were more prominent than unfocused subjects or objects in terms of all four parameters (all *p*-values < 0.05). Across sentence types, subject words in the subject-stressed sentence type (ScleftS) had longer duration, higher F0, larger F0 range, and higher intensity than those in the object-stressed sentence type (canonO and ScleftO) (all *p*-values < 0.05). Moreover, object words in ScleftS were less prominent than those in canonO and ScleftO (all *p*-values < 0.05). The aforementioned differences confirm that the materials were created as intended.

**Table 2:** Fitted mean values of duration (ms), F0 (Hz), F0 range (Hz), and intensity (dB) of subject and object nouns in Chinese critical stimuli.

**Sentence condition**	**Word position**	**Duration**	**F0**	**F0 range**	**Intensity**
canonO	Subject	566	216	81	70
	Object	740	288	243	75
ScleftO	Subject	535	210	72	70
	Object	732	283	243	75
ScleftS	Subject	680	336	264	79
	Object	585	180	85	64

**Table 3:** The ANOVA tables for duration, F0, F0 range, and intensity analysis.

	**Chisq**	**Df**	***P***
**Duration: [model:duration** ~ SentenceType^*^wordPosition+			
**ToneCombination+(1|word)]**			
SentenceType	15.87	2	<0.001
WordPosition	103.56	1	<0.001
ToneCombination	40.01	19	0.003
SentenceType:wordPosition	738.84	2	<0.001
**F0: [model:F0** ~ SentenceType^*^wordPosition+ToneCombination+(1|**word)]**			
SentenceType	18.02	2	<0.001
WordPosition	0.24	1	0.621
ToneCombination	139.99	19	<0.001
SentenceType:wordPosition	1781.49	2	<0.001
**F0 range: [model:F0 range**~ SentenceType^*^wordPosition+			
**ToneCombination+(1|word)]**			
SentenceType	6.64	2	0.036
WordPosition	45.57	1	<0.001
ToneCombination	70	19	<0.001
SentenceType:wordPosition	666.54	2	<0.001
**Intensity: [model:intensity** ~SentenceType^*^wordPosition+(1|**word)]**			
SentenceType	32.70	2	<0.001
WordPosition	6.51	1	0.011
SentenceType:wordPosition	2950.44	2	<0.001

Stimulus onset asynchrony (SOA), the duration between the offset of the prime word and the onset of the visual target, was shown to influence the priming of target words in Husband and Ferreira (2016), compared to no SOA (0 ms). In order to keep the SOA constant, a variable duration of silence (0 ms to 607 ms) was added to the end of each sound file, so that the SOA was always 1,500 ms.

##### 4.2.2.4. Other items

A further 150 filler sentences with word and non-word targets were constructed, which lead to a total of 210 trials per list (60 test items + 150 fillers). As the experiment task is to decide whether two characters make up a real word or not in Mandarin Chinese, we included non-words to avoid response bias. Among these filler targets, 105 were non-words and 45 were words to counterbalance yes/no responses across the whole experiment. Sixty of the filler sentences had the same sentence types (canonO, ScleftO, ScleftS) with non-words as target words. Among the non-words, ten were phonologically related to one of the words in the sentence to encourage different types of priming. Another 90 filler sentences with different sentence structures (SV, SVAdv etc.) were also constructed, including 45 sentences with words and 45 with non-words as visual targets. Ten words and 10 non-words were phonologically related to one of the words in the sentence. Non-words were selected from the lexical decision data from Cai and Brysbaert (2010) with 100% non-word accuracy. The non-words consist of two real characters which do not make up a real word together. Six practice sentences which had three word and three non-word visual targets were also prepared. Furthermore, 12 comprehension questions asking the content of a previous filler were included to encourage participants to pay attention to the sentences.

#### 4.2.3. Procedure

The experiment was administered using Opensesame v. 3.1 (Mathôt et al., 2012), and was run in a quiet computer room at Henan Polytechnic University. The entire session was conducted in Chinese. Participants were seated in front of a computer screen with a closed-ear headphone. At the start of the experiment, participants received written instructions on the computer screen, and the instructions were also repeated orally by the experimenter (first author) after the participants had read them. In the practice phase, participants first heard a sentence, and while the sentence was being played, participants concentrated on a fixation dot in the middle of the screen. Then they saw two characters, and had to decide whether these two characters made up a real word or not by pressing “m” key [labeled as 是 (“yes”)] for *yes* response and “z” key [labeled as 否 (“no”)] for *no* response using their dominant hand as fast as they could. In the practice phase, participants received feedback on their responses (if their answer was wrong) and reaction times (RTs) (if their response time exceeded 1,000 ms).

The procedure of the main experiment was similar to the practice phase, but no feedback was provided. The main experiment moved to the next trial automatically if no key was pressed within 3 s. The stimuli were divided into four blocks with a 10 s compulsory break, or longer if participants wanted, between two blocks. The stimuli within a block were randomized as well as the order of blocks. Twelve filler trials were followed by the twelve comprehension questions which appeared randomly and evenly across the four blocks. The comprehension questions required “x” or “n” key press to adjust to the comprehension questions being a different task (from lexical decision) and therefore avoid mistakes. There was always a filler trial following the comprehension question. The entire experiment lasted approximately 15 minutes. Demographic information such as sex, age, hometown, and English proficiency was collected using a paper form at the end of the experiment.

#### 4.2.4. Analysis Method

Both accuracy and response times (RTs) were measured. The accuracy measure enabled us to look at whether different focus conditions and target types had any influence on the difficulty of the lexical decision. RTs of lexical decisions reflected the activation of the visual target word by the auditory prime sentence. As priming was of central interest in the study, we primarily looked at the RTs. The comparison of RTs to the related words (identical, contrastive, non-contrastive) to unrelated baseline controls shows whether the related words were primed or not.

Mixed effects regression models were built to test how the accuracy and RTs were affected by a number of factors, using the R package lme4 (Bates et al., 2015). For the accuracy analysis, response choice was the dependent variable in generalized linear mixed effects models (family: binomial) and for the RT analysis, reaction times were the dependent variable in linear mixed effects regression.

The fixed effects of the initial model included key experimental predictors and item factors. The key experimental predictors were sentence type (canonO, ScleftO, ScleftS) and target type (identical, contrastive, non-contrastive, and control). Backward difference coding was used to better represent the internal structure of sentence types, resulting in two variables: syntax (canonO vs. ScleftO) and prosody (ScleftO vs. ScleftS). The item factors included the log frequency of target words, the centered position of the trial in the experiment, and the transformed RTs of the previous trial, as these factors have been previously shown to influence RTs (e.g., Braun and Tagliapietra, 2010; Gotzner, 2017). Similarly, whether the previous target was a word and whether the previous response was correct were included as they can have spillover effects on the subsequent trial. Silence duration was also included as a predictor in the model, as this was variable between stimuli^2^.

In addition to the fixed effects, the random effects, motivated by the literature and justified by the data, included intercepts for participants and target words, random slopes for trial (position in the experiment) by participants and by items and random slopes for the interactions between the key experimental factors by participants and by items. If the initial model did not converge, the model was simplified by reducing random structures, i.e. taking out the slopes that had the lowest variance scores until the model converged. When the model converged, the step function in the lmerTest package (Kuznetsova et al., 2017) was used to eliminate non-significant fixed and random effects. Only the factors that significantly contributed to the model fit were kept in the final model.

### 4.3. Results

A total of 20,790 responses were recorded, 210 from each of 99 participants. The overall accuracy is 91.5% for responses and 98.2% for comprehension questions. Data from three participants was excluded for low accuracy on target word responses, and one further as the “yes” button was not pressed with the dominant hand. The remaining 5,700 critical trials from 95 participants were used for accuracy analysis. A further 123 trials with incorrect responses (2.2%) were excluded, leaving 5,577 for the response time analysis. Further, data points of residuals whose standard deviations were larger than 2.5 were eliminated. The RTs were inverse transformed, which was the best transformation (that had the highest correlation in a quantile-quantile plot of the distribution), compared with no transformation, log transformation and inverse square root transformation. The transformed RTs were then multiplied by 10,000 in order to make the estimates and SD more readable.

#### 4.3.1. Accuracy

The overall accuracy on the experimental trials was 97.8%. Mixed effect logistic regression models were built to test the factors affecting accuracy, following the process detailed above in section 4.2.4. The final model did not include sentence type, and had a random effect of Participant only. The fitted accuracy was 99.1% for identical, 98.9% for contrastive, 98.6% for non-contrastive, and 97.8% for controls. The ANOVA table of the final model showing the significance of the fixed effects is in Table 4. Participants were more accurate for more frequent targets (β = 0.73, SD = 0.22) and later in the experiment (β = 0.01, SD < 0.01). In order to test which target types differed from each other, we conducted planned comparisons using the glht function in the multcomp package (Hothorn et al., 2008). Identical and contrastive items received higher accuracy rates than control items (identical: *z* = 3.1, *p* = 0.01; contrastive: *z* = 2.8, *p* = 0.03), but no significant differences were found between other target types (identical vs. contrastive; identical vs. non-contrastive; contrastive vs. non-contrastive; all *p*-values > 0.1).

**Table 4:** Fixed effects of mixed effects model with accuracy or transformed reaction times as the dependent variable.

	**Chisq**	**Df**	***P***
**Accuracy: [model:correct ~ TargetType+log frequency+centerd trial**			
**+(1|Participant)]**			
TargetType	13.34	3	<0.001
log frequency	11.12	1	<0.001
centered trial	10.25	1	0.001
RTs: [model:transformed RTs ~ Sentence condition^*^TargetType			
**+log frequency+centerd trial+PreCorrectness+PreRT+PreWordness**			
**+(1|Participant) + (1|Item)]**			
Sentence condition	18.11	2	<0.001
TargetType	49.38	3	<0.001
log frequency	36.21	1	<0.001
centerd trial	90.99	1	<0.001
PreCorrectness	9.05	1	0.003
PreRT	181.82	1	<0.001
PreWordness	23.96	1	<0.001
Sentence condition:TargetType	14.96	6	0.021

#### 4.3.2. Reaction Times

Mixed effect linear regression models were built to test the factors affecting RTs, following the process detailed in section 4.2.4. The ANOVA table showing the significance of variables in the final model is given in Table 4. The final model had random effects for Participant and Item. Participants became faster over the course of the experiment (centered trial: β = 0.006, SD = 0.001). Since the dependent variable is an inverse transform of RT, negative coefficient estimates represent slower responses, and positive coefficient estimates represent faster responses. Words of higher frequency were recognized faster (log frequency: β = 0.82, SD = 0.14). Participants responded more quickly when the previous response was correct (PreCorrectness: β = 0.57, SD = 0.19); and when the previous trial was a word (PreWordness: β = 0.39, SD = 0.08). Participants responded more slowly when the transformed RT to the previous trial was slow (PreRT: β = –0.003, SD = < 0.001). None of the other factors included in the initial model were significant (see section 4.2.4), thus we will not discuss them.

**Figure 3:**
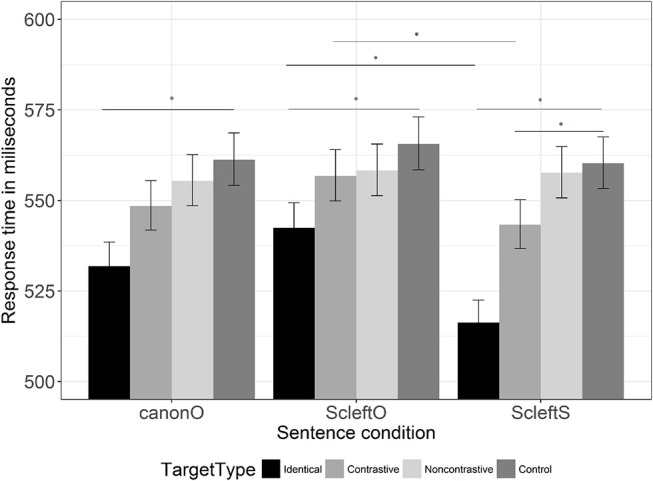
Back-transformed fitted RTs in ms to four target types in canonO, ScleftO, and ScleftS conditions. Error bars show standard error of the means. Stars (^*^) show significant comparisons (*p* < 0.05).

The final model showed main effects of sentence condition and target type, as well as their interaction (see Table 4). The fitted RTs are shown in Figure 3. As expected, identical words were recognized the fastest at 530 ms, then contrastive alternatives (549.5 ms), and then the other two target types (non-contrastive: 557.1 ms; control: 562.4 ms). For sentence condition, ScleftS was the fastest (543.1 ms), followed by canonO (548.3 ms) and ScleftO (555.6 ms). In order to find out how different target types were affected by sentence condition, we conducted planned comparisons on the interaction using the glht function in the multcomp package. In order to run the comparison, the model was rerun with the interaction between sentence condition and target type as a single factor.

To investigate the first research question: whether prosodic or syntactic F-marking is necessary for subject nouns to prime *themselves*, we conducted planned comparisons between identical items (subject nouns) and unrelated controls in the no F-marking condition (canonO), the syntactic F-marking condition (ScleftO) and the prosodic+syntactic F-marking condition (ScleftS). Identical items showed facilitation over unrelated controls in all sentence conditions (canonO: *z* = 4.37, *p* < 0.001; ScleftO: *z* = 3.37, *p* = 0.003; ScleftS: *z* = 6.79, *p* < 0.001). This indicates that F-marking is not necessary for subject nouns to prime themselves, as subjects nouns were recognized faster than unrelated controls in the no F-marking condition.

To investigate the second research question: whether prosodic or syntactic F-marking is necessary for subject nouns to prime their *contrastive alternatives*, we conducted planned comparisons between contrastive alternatives and unrelated controls in the three focus conditions. Contrastive alternatives were facilitated over unrelated controls in the ScleftS condition (ScleftS: *z* = 2.5, *p* = 0.043), but not in the other two conditions (canonO: *z* = 1.86, *p* = 0.135; ScleftO: *z* = 1.25, *p* = 0.358). This shows that prosodic F-marking is necessary for subject nouns to prime contrastive alternatives, as contrastive alternatives were only recognized faster than unrelated controls in the ScleftS condition.

To investigate the third research question: whether prosodic or syntactic F-marking is necessary for subject nouns to prime their *non-contrastive associates*, we also conducted planned comparisons between non-contrastive associates and unrelated controls in the three focus conditions. None of the comparisons were significant (all *p*-values > 0.1). This showed that the non-contrastive associates were not primed in any of the conditions.

We also did planned comparisons between the syntactic F-marking condition and the no F-marking and the prosodic+syntactic F-marking condition (canonO vs. ScleftO; ScleftS vs. ScleftO) for all four target types. The results showed that only identical items and contrastive alternatives were facilitated in the prosodic+syntactic condition compared to the syntactic condition, which showed that prosodic F-marking strengthened the priming of identical items (*z* = 4.96, *p* < 0.001), and that prosodic F-marking primed contrastive alternatives (*z* = 2.4, *p* = 0.047). All the other comparisons were not significant (all *p*-values > 0.1).

Table 5 summarizes the comparisons laid out above that were relevant and important to answer the research questions, e.g., identical words were primed (relative to unrelated controls) in all sentence conditions, and the priming was strengthened in the ScleftS condition. Contrastive alternatives were primed in the ScleftS condition, but not in canonO and ScletO conditions. Non-contrastive associates were not facilitated over unrelated controls in all sentence conditions.

**Table 5:** Comparisons of related words (identical, contrastive, non-contrastive) and unrelated controls in all three sentence conditions (canonO, ScleftO, ScleftS).

**Target types**	**canonO (no F-marking)**	**ScleftO (syntactic F-marking)**	**ScleftS (syntactic + prosodic F-marking)**
Identical vs. control	^*^	^*^	^*^
Contrastive vs. control	NS	NS	^*^
Non-contrastive vs. control	NS	NS	NS

Star(

* *)show significant comparisons (p < 0.05)*.

We also ran an additional analysis to test the effects of the relatedness of the prime word to the visual target, using the relatedness scores from our questionnaire (see section 4.2.2). This analysis excluded trials with identical targets, as this would be between a prime word and itself. An ANOVA model comparison showed that relatedness did not significantly improve the model fit[$\chi_{\left(1\right)}^{2}$=2.04, *p* = 0.15].

## 5. General Discussion

We reported a cross-modal lexical decision experiment, looking at the priming of different kinds of targets in Mandarin Chinese. Primes were subject nouns in spoken sentences. Targets were presented after the sentences, with a fixed SOA of 1,500 ms. The experiment looked at four target types: identical items, contrastive alternatives, non-contrastive associates, and unrelated controls; and three sentence types: no focus-marking (canonO, canonical order with nuclear stress on the object), syntactic focus-marking (ScleftO, subject cleft with nuclear stress on the object), or prosodic+syntactic focus-marking (ScleftS, subject cleft with nuclear stress on the subject). The study addressed three main questions (see section 4.1): whether prosodic or syntactic focus-marking is necessary for subject nouns to prime *themselves, their contrastive alternatives*, and *non-contrastive associates*.

In relation to the first research question, subject nouns in spoken sentences prime themselves in Mandarin Chinese (identity priming). Identical words were responded to significantly faster than unrelated controls in all conditions. Further, the priming effect was strengthened by prosodic focus-marking (see Figure 3). This is consistent with the effect of prosodic focus-marking, in the absence of syntactic focus-marking (i.e., canonS vs. canonO), on identical priming reported in Yan et al. (2019). There it was also found that identical items were responded to faster when they were prosodically prominent (prosodic focus-marking). This shows that focus-marking is not necessary for subject nouns to prime themselves; however prosodic focus-marking, but not syntactic focus-marking, strengthens the priming. The general result that identity priming is found in all focus conditions is consistent with Norris et al. (2006) for English, and validates the effectiveness of the methodology in Chinese. Together with our results reported in Yan et al. (2019), this shows for the first time that identity priming is strengthened by prosodic focus-marking in Chinese.

In relation to the second research question, contrastive alternatives were recognized significantly faster than unrelated controls in the prosodic+syntactic focus-marking condition, but not in the no focus-marking and syntactic focus-marking conditions. Therefore, prosodic focus-marking is necessary for subject nouns to prime their contrastive alternatives, which is consistent with the findings in Yan et al. (2019) in the absence of syntactic focus-marking in Mandarin. This is also consistent with what Braun and Tagliapietra (2010) found for Dutch, but is different to what Husband and Ferreira (2016) found for English, who found that contrastive alternatives were responded to faster than controls in both the neutral and contrastive accenting conditions in English (with an SOA of 750 ms, which is closest to our experiment). In our study, syntactic focus-marking did not play a similar role in the priming of contrastive alternatives, as contrastive alternatives were not recognized faster than unrelated controls when the subject nouns were marked with syntactic focus-marking.

In relation to the third research question, the RTs of non-contrastive associates were not significantly different from those of unrelated controls in any sentence condition, nor did the RTs for non-contrastive associates significantly differ by sentence condition, showing they were not primed. RTs for non-contrastive associates were, however, numerically faster than for controls across conditions. This result is again consistent with that found in Yan et al. (2019), which also showed no difference in RTs between non-contrastive associates and unrelated controls regardless of prosodic focus-marking (canonS vs. canonO). Concerning the role of prosodic focus-marking in priming non-contrastive associates, this result is largely consistent with Braun and Tagliapietra (2010), who found weak priming of non-contrastive associates regardless of sentence conditions in Dutch. Even though non-contrastive associates had only numerical facilitation with prosodic focus-marking in our study and Husband and Ferreira, our finding is different from Husband and Ferreira (2016), who only found priming of non-contrastive associates in later processing (SOA 750 ms) in their neutral accent condition, but no priming of non-contrastive associates with prosodic focus-marking.

These results therefore provide cross-linguistic psycholinguistic evidence for the role of prosodic focus-marking in lexical activation. They extend previous findings, using phoneme monitoring and memory tasks, that prosodic focus-marking increases attention to and activation of the focused word, by showing prosodic focus-marking strengthens identity priming. Further, together with the results in Yan et al. (2019), they show for the first time in a non-Germanic language, evidence for prosodic focus-marking as activating alternatives to the focused word, consistent with Rooth's (1992) theory, by showing that prosodic focus-marking primes contrastive alternatives to subject nouns in Chinese, in canonical order and cleft sentences. These findings are consistent with those for Dutch reported in Braun and Tagliapietra (2010), and related findings using eye-tracking and other findings in Germanic languages reported in section 3. The differences between contrastive and non-contrastive associates in the Chinese experiment and earlier studies show this is not a general semantic priming effect, but is rather consistent with the role of prosodic focus-marking in triggering an implication of alternatives.

Our results on the role of prosodic focus-marking in priming contrastive alternatives are consistent with those found for Dutch by Braun and Tagliapietra (2010), in that contrastive alternatives were only primed with contrastive prosody, but are different from those found for English by Husband and Ferreira (2016), who found priming of contrastive alternatives with neutral or contrastive accenting. There were some methodological differences between the earlier studies and ours, e.g., in how semantic relatedness between target types was controlled, and in relation to the timing of the presentation of the targets (SOA). These were presented immediately after the object prime in Braun and Tagliapietra (2010), with an SOA of both 0 ms and 750 ms in Husband and Ferreira (2016), and with an SOA of 1,500 ms in this study. The time course therefore does not seem to account for the difference in results for priming of contrastive alternatives, but rather suggests that contrastive alternatives remain activated for a long time course. This is consistent with the facilitation results in memory tasks reported in section 3. There were also differences between the studies in the prosodic realization of the “neutral” or “no prosodic marking” condition. In both Braun and Tagliapietra (2010) for Dutch and Husband and Ferreira (2016) for English, the prime word in their “neutral” accent condition was in fact still accented. In Dutch, this was an !H^*^ accent at the end the Dutch “hat pattern”, with steady, low or falling pitch through the object word; whereas in English, the (!)H^*^ accent was a definite rise, although small. Thus, the former may have been less prosodically prominent than the latter. In the Chinese stimuli for canonO and ScleftO, the pitch range was relatively narrow for the subject, and much wider for the object. Therefore, we speculate that the Chinese and Dutch “neutral”/“no focus” stimuli were more similar in terms of prosodic realization.

On the other hand, our results in relation to non-contrastive associates were more similar to Husband and Ferreira (2016). Braun and Tagliapietra (2010) found weak priming of non-contrastive associates regardless of prosody at 0 ms SOA, and Husband and Ferreira (2016) found priming only with contrastive accenting at 0 ms, and only without contrastive accenting at 750 ms, while we found no priming, regardless of prosody, at 1,500 ms SOA. In this case, the time course of presentation does seem like the most likely reason for the differences in results. As discussed in section 3, general semantic priming is not consistent, and it may be shorted-lived (see e.g., Neely, 1977). Husband and Ferreira (2016) account for their results in terms of rapid decay of general (non-contrastive) semantic associates, which is expedited by contrastive accenting. Considering our SOA was even longer (1,500 ms), it may be that general semantic priming had decayed over this time course, regardless of prosody. It is also possible that these different findings for contrastive and non-contrastive associate priming stem from language-specific differences in processing, though there is no obvious reason for the particular differences between Dutch, English, and Chinese found.

In Chinese, syntactic focus-marking without prosodic prominence (ScleftO) seemed to slow recognition times in general; although the differences were not significant. One reason might be that ScleftO sentences usually require a context, such as (4a) in section 2, where the subject is presupposed. The relative unusualness out of context might have slowed responses. These findings resemble those in Gotzner (2017), who found the exclusive focus particle *only* also slowed listeners' response times. She argued that focus particles had interference effects caused by stronger competition among members of the alternative set. Similarly, here responses could be slowed by the difficulty of encoding the presuppositions required by the ScleftO structure. On the other hand, Gotzner showed that in memory, *only* had a processing advantage. It could be that more complex ways of marking focus have an immediate processing cost, but a later processing advantage. In future work it would be good to look at the effect of syntactic focus in memory tasks.

What does this mean for the relationship between different types of focus-marking and lexical activation, given that we have found that prosodic focus-marking, but not syntactic focus-marking, strengthens activation of focused words and is necessary for alternative priming in Chinese? If focus-marking, and not specifically prosodic focus-marking, is the underlying mechanism, it is surprising that syntactic focus-marking did not strengthen priming. Perhaps contrastive prosodic prominence is the underlying mechanism, and the focus effect observed in previous studies with written syntactic clefting may be triggered by the implicit contrastive prominence. Therefore, it is possible that the activation is rather related to prosodic prominence, which enhances the salience of the prominent word, and therefore its processing, rather than focus-marking. However, it is also possible that prosodic and syntactic focus-marking play different roles, at least in Germanic and Chinese. As discussed above, syntactic clefting (and other morphosyntactic markers like focus particles) carry additional implications, such as more complex presuppositions and exhaustivity. This may slow processing in the short term, but have memory advantages. A further reason for the finding could be because, in Chinese (and Germanic), prosody is the primary cue to focus, while syntax is secondary (and hence carries additional implications). If we were to look at languages where morphosyntactic markers were the primary cue to focus, and prosody secondary, we would expect to see strengthening of activation and priming of alternatives given those morphosyntactic markers. We need more studies to distinguish between these possible explanations, but these results suggest this is a fruitful area for future research.

This study aimed to shed light on the role of focus-marking in lexical activation, and particularly, in the linguistic cues which listeners use to activate alternatives in spoken sentences. This is an important part of understanding the processes by which listeners understand implicatures related to alternatives in speech. The results further strengthened earlier findings for the importance of prosodic prominence in strengthening activation of focused words and their alternatives, and importantly, provided cross-linguistic validation of this in Chinese. However, it revealed a complex picture of the cues which strengthen identity priming and trigger alternative priming, i.e., prosody but not necessarily syntax. We hope this will prompt more work on the linguistic cues to focus listeners attend to in speech, and their apparently highly contextual nature.

## Ethics Statement

This study was carried out in accordance with the recommendations of Victoria University of Wellington Human Ethics Committee with written informed consent from all subjects. All subjects gave written informed consent in accordance with the Declaration of Helsinki. The protocol was approved by the Victoria University of Wellington Human Ethics Committee.

## Author Contributions

This study was originally conceived by SC. The experiment was mainly designed and built by MY with contributions by SC. Both authors contributed to the data collection and data analysis and the write up of the paper.

### Conflict of Interest Statement

The authors declare that the research was conducted in the absence of any commercial or financial relationships that could be construed as a potential conflict of interest.

## Footnotes

^1^The first tier of (1b) indicates the Chinese characters. The second tier shows Pinyin, which is the official romanisation of Chinese. The numbers (0–4) represent tones (neutral, high, rising, low, falling). In the third tier, the following abbreviations are used in glosses: PRF = perfective aspect, COP, copula; S, subject; V, verb; O, object. 的 (*DE*) is glossed as *DE* following the current literature such as Paul and Whitman (2008) and Hole (2011). Note that Mandarin Chinese 的 (*DE*) has multiple uses (apart from its association with past tense reading in this paper), which includes its function as a complementizer, a nominalizer and others (see e.g., Paul and Whitman, 2008; Xie, 2012).

^2^Note that silence duration was correlated with sentence type, as canonO and ScleftO sentences were longer than ScleftS sentences because of the stress on the object noun. We therefore initially regressed silence duration against sentence type [*F*_(2)_ = 2710, *p* < 0.001]. The residuals (*Residual Silence*) (the difference from the mean silence duration for each sentence type) were used as a predictor. But one reviewer pointed out the issues with residualization as a way to deal with colinearity (see Wurm and Fisicaro, 2014). We therefore used the unresidualized variable in the model. It should be noted that neither residualized nor unresidualized variable significantly improved the model fit (*p* > 0.1), so we did not keep it in the final model.

## Supplementary Material

The sound files for the eight examples shown in Figures 1 and 2 are included as supplemental material, along with the full list of stimuli, relatedness scores, and word frequencies for the experiments.
