# Supplementary material for: Priming Effects of Focus in Mandarin Chinese
Source: Front Psychol. 2019 Aug 30;10:1985. doi: 10.3389/fpsyg.2019.01985 (PMC6730480; doi:10.3389/fpsyg.2019.01985)
Supplement: Supplementary file 2 [file Data_Sheet_2.pdf]

Supplementary Material: What primes alternatives? Investigating syntactic and prosodic focus priming of alternatives in Mandarin Chinese and English

1 EXPERIMENTAL STIMULI

The prime word is the subject noun. Only the canonical sentences are listed here.

No.	Sentence	Subject	Alternative	Noncontrastive	Unrelated
1	婴儿吃完了饼干	婴儿	保姆	摇篮	武器
	The infant finished the biscuits	infant	nanny	cradle	weapon
	frequency	3.15	3.05	2.05	3.56
	relatedness		4.83	5.29	1.46
2	香蕉摆满了货架	香蕉	苹果	猴子	天空
	The banana occupied the shelf	banana	apple	monkey	sky
	frequency	2.60	2.88	2.95	2.87
	relatedness		4.83	4.13	1.46
3	海滩迷住了姐姐	海滩	灯塔	假期	绷带
	The beach captivated my sister	beach	lighthouse	holiday	bandage
	frequency	3.04	2.26	2.77	2.26
	relatedness		4.48	4.50	1.46
4	蛋糕招来了虫子	蛋糕	面包	生日	邮件
	The cake attracted insects	cake	bread	birthday	mail
	frequency	3.30	3.08	3.58	3.08
	relatedness		4.88	6.15	1.60
5	船长穿上了雨衣	船长	水手	甲板	南瓜
	The captain put on the raincoat	captain	sailor	deck	pumpkin
	frequency	3.14	2.47	2.45	2.48
	relatedness		5.58	4.58	1.35
6	香槟洒满了桌子	香槟	啤酒	法国	角色
	The champagne spilled over the table	champagne	beer	France	role
	frequency	2.91	3.36	3.32	3.38
	relatedness		5.00	4.75	1.76
7	咖啡弄脏了衬衣	咖啡	牛奶	早晨	卫星
	The coffee dirtied the shirt	coffee	milk	morning	satellite
	frequency	3.62	2.95	3.01	2.95
	relatedness		4.71	2.83	1.15
8	硬币掉进了口袋	硬币	金子	银行	例子
	The coin fell into the pocket	coin	gold	bank	example
	frequency	2.76	2.63	3.48	2.61

	relatedness		4.52	4.25	1.43
9	玉米掉出了篮子	玉米	花生	农场	脉搏
	The corn fell out of a basket	corn	peanut	farm	pulse
	frequency	2.82	2.48	2.99	2.49
	relatedness		4.15	5.04	1.24
10	乌鸦躲进了草丛	乌鸦	鸽子	羽毛	股票
	The crow hid in the grass	crow	pigeon	feather	stock
	frequency	2.29	2.67	2.35	2.68
	relatedness		4.13	4.38	1.42
11	顾客关上了窗户	顾客	店主	产品	陆地
	The customer closed the window	customer	shop owner	product	land
	frequency	2.99	2.17	2.94	2.16
	relatedness		5.07	5.21	2.14
12	女儿喝完了果汁	女儿	侄子	裙子	炸药
	The daughter finished the juice	daughter	nephew	dress	explosive
	frequency	3.93	2.67	3.07	2.65
	relatedness		3.67	4.56	1.00
13	晚餐提供了可乐	晚餐	早饭	日落	地震
	The dinner came with the coke	dinner	breakfast	sunset	earthquake
	frequency	3.38	2.69	2.54	2.69
	relatedness		5.44	3.67	1.48
14	英语打击了张三	英语	数学	字母	地盘
	English discouraged Zhangsan	English	math	letter	domain
	frequency	3.07	2.87	2.83	2.86
	relatedness		4.54	5.21	1.62
15	模特赢得了冠军	模特	明星	身材	信号
	The model won the first prize	model	celebrity	body shape	signal
	frequency	3.47	3.39	2.86	3.35
	relatedness		4.00	6.07	1.92
16	蜜蜂闯进了厕所	蜜蜂	蝴蝶	鲜花	小费
	The bee broke into the toilet	bee	butterfly	flower	tip
	frequency	2.76	2.59	2.39	2.60
	relatedness		4.33	5.67	1.38
17	苍蝇弄伤了眼睛	苍蝇	蚊子	垃圾	深渊
	The fly hurt the eyes	fly	mosquito	garbage	abyss
	frequency	2.46	2.11	3.40	2.12
	relatedness		4.79	3.92	1.19
18	围巾塞满了书包	围巾	袜子	脖子	闪电
	The scarves occupied the school bag	scarf	sock	neck	lightning
	frequency	2.35	2.72	3.11	2.73
	relatedness		3.70	5.08	1.76
19	国王写完了日记	国王	皇后	皇冠	馅饼
	The king finished writing the diary	king	queen	crown	pie
	frequency	3.25	2.80	2.31	2.80
	relatedness		5.50	5.52	1.55

20	律师摔碎了杯子	律师	法官	法院	生意
	The lawyer smashed the glass	lawyer	judge	court	business
	frequency	3.83	3.57	2.94	3.55
	relatedness		5.17	5.25	3.31
21	经理租借了相机	经理	秘书	职位	隧道
	The manager rented the camera	manager	secretary	position	tunnel
	frequency	3.24	2.74	2.82	2.71
	relatedness		4.63	5.59	1.45
22	镜子弄破了手指	镜子	玻璃	图像	数据
	The mirror cut the finger	mirror	glass	image	data
	frequency	2.89	3.05	2.68	3.05
	relatedness		4.96	4.08	1.42
23	报纸掉进了袋子	报纸	杂志	新闻	英雄
	The newspaper fell into the bag	newspaper	magazine	news	hero
	frequency	3.23	3.38	3.54	3.44
	relatedness		5.30	5.88	2.10
24	小说感动了张三	小说	诗歌	版权	坚果
	The fiction moved Zhangsan	novel	poetry	copyright	nut
	frequency	2.99	2.24	1.88	2.24
	relatedness		4.33	4.58	1.23
25	乘客捡到了钱包	乘客	司机	公交	天堂
	The passenger found the wallet	passenger	driver	bus	heaven
	frequency	2.79	3.19	2.30	3.18
	relatedness		4.54	4.54	1.62
26	病人吃完了葡萄	病人	护士	诊所	环境
	The patient ate up the grapes	patient	nurse	clinic	environment
	frequency	3.53	3.14	2.86	3.12
	relatedness		5.29	5.56	3.90
27	博士阅读了散文	博士	教授	学位	机器
	The Ph.D. student read the novel	PhD	professor	degree	machine
	frequency	3.69	3.28	2.55	3.23
	relatedness		4.79	5.50	2.08
28	钢琴吵醒了叔叔	钢琴	吉他	节奏	海岸
	The piano woke (my) uncle up	piano	guitar	rhythm	coast
	frequency	2.87	2.85	2.69	2.86
	relatedness		4.79	4.58	1.89
29	手枪吓坏了青蛙	手枪	子弹	军事	答案
	The gun scared the frog	gun	bullet	military affair	answer
	frequency	2.79	3.40	2.80	3.45
	relatedness		6.15	5.04	2.26
30	裁判出席了会议	裁判	教练	规则	公园
	The referee attended the meeting	referee	coach	rule	park
	frequency	2.60	3.31	3.19	3.34
	relatedness		4.04	5.54	1.29
31	餐馆雇佣了会计	餐馆	酒店	食物	颜色

	The restaurant hired the accountant	restaurant	hotel	food	color
	frequency	3.06	3.31	3.41	3.23
	relatedness		4.81	5.96	2.00
32	戒指装满了抽屉	戒指	项链	左手	广场
	The rings filled up the drawer	ring	necklace	left hand	square
	frequency	3.20	2.83	2.54	2.82
	relatedness		4.58	4.21	1.42
33	室友打破了茶杯	室友	同学	宿舍	电梯
	The roommate broke the cup	roommate	classmate	dorm	lift
	frequency	2.82	3.01	2.53	3.01
	relatedness		4.83	5.93	1.65
34	玫瑰划破了大腿	玫瑰	树叶	花园	快餐
	The roses pierced my thigh	rose	leaves	garden	fast food
	frequency	2.77	2.10	2.87	2.10
	relatedness		3.88	4.38	1.54
35	香肠引来了狐狸	香肠	热狗	脂肪	奖金
	The sausage attracted the fox	sausage	hot dog	fat	bonus
	frequency	2.60	2.75	2.42	2.75
	relatedness		4.92	3.89	2.00
36	海豹抢到了气球	海豹	海豚	海洋	海关
	The seal grabbed the balloon	seal	dolphin	sea	Customs
	frequency	2.16	2.32	2.66	2.23
	relatedness		3.92	4.21	1.46
37	歌手收养了孤儿	歌手	画家	专辑	草坪
	The singer adopted the orphan	singer	painter	album	lawn
	frequency	3.14	2.47	2.76	2.47
	relatedness		3.89	5.50	1.81
38	士兵弄丢了钥匙	士兵	将军	战争	太阳
	The soldier lost the key	soldier	General	war	sun
	frequency	3.21	3.33	3.49	3.15
	relatedness		5.26	5.79	1.67
39	间谍拿走了芯片	间谍	侦探	阴谋	舞会
	The spy took the chip	spy	detective	conspiracy	ball
	frequency	3.11	3.27	2.79	3.26
	relatedness		4.92	4.38	2.27
40	风暴摧毁了植物	风暴	大雪	气象	蜡笔
	The storm destroyed the plants	storm	heavy snow	meteorology	crayon
	frequency	2.70	1.70	2.13	1.70
	relatedness		4.33	5.13	1.31
41	老师喝完了绿茶	老师	校长	小学	太空
	The teacher finished the green tea	teacher	principal	primary school	space
	frequency	3.59	2.97	2.53	2.97
	relatedness		5.29	3.67	1.38
42	老虎喝完了雨水	老虎	狮子	笼子	月亮
	The tiger drank up the rain water	tiger	lion	cage	moon

	frequency	2.57	2.63	2.53	2.63
	relatedness		4.25	3.33	1.45
43	电视预报了小雨	电视	广播	屏幕	码头
	TV forecast light rain	TV	broadcast	screen	pier
	frequency	3.68	2.87	2.68	2.85
	relatedness		4.63	5.85	1.95
44	大学雇佣了哥哥	大学	高中	专业	舞台
	The university hired (my) brother	university	high school	major	stage
	frequency	3.65	3.33	3.21	3.28
	relatedness		5.74	4.96	3.19
45	蔬菜堆满了仓库	蔬菜	水果	营养	路线
	The vegetables occupied the storage room	vegetables	fruits	nutrition	route
	frequency	2.54	2.77	2.33	2.78
	relatedness		4.96	5.00	1.85
46	病毒引发了头痛	病毒	细菌	细胞	标签
	The virus caused the headache	virus	bacteria	cell	tag
	frequency	3.05	2.53	2.68	2.53
	relatedness		5.33	5.04	2.14
47	妻子购买了手表	妻子	丈夫	女性	节目
	The wife bought the watch	wife	husband	female	program
	frequency	3.81	3.79	3.32	3.80
	relatedness		5.83	5.67	1.95
48	胳膊碰倒了花瓶	胳膊	肩膀	肌肉	沙漠
	The arm knocked the vase over	arm	shoulder	muscle	desert
	frequency	2.93	2.84	2.89	2.83
	relatedness		4.96	4.89	1.10
49	篮球砸伤了张三	篮球	足球	球场	月球
	The basketball hit and hurt Zhangsan	basketball	football	ball field	moon
	frequency	2.87	2.79	2.62	2.49
	relatedness		4.00	5.78	2.29
50	卧室传出了笑声	卧室	客厅	枕头	轨道
	There were laughs coming from the bedroom	bedroom	living room	pillow	track
	frequency	3.00	2.58	2.69	2.59
	relatedness		5.19	5.08	1.76
51	老板捡起了橡皮	老板	同事	公司	阳光
	The boss picked up the eraser	boss	colleague	company	sunlight
	frequency	3.52	3.14	4.00	3.05
	relatedness		4.71	5.81	2.19
52	电脑损伤了视力	电脑	手机	网站	城市
	Computers damaged the eyesight	computers	mobile phone	website	city
	frequency	3.51	3.56	2.92	3.52
	relatedness		4.25	5.70	2.76
53	书桌压住了毛巾	书桌	椅子	功课	癌症

	The desk scrooched the towel	desk	chair	homework	cancer
	frequency	1.89	3.07	2.44	3.02
	relatedness		5.13	4.38	1.08
54	夹克阻挡了大雨	夹克	裤子	纽扣	偶像
	The jacket blocked the rain	jacket	pants	button	idol
	frequency	2.74	3.27	2.20	3.27
	relatedness		3.92	3.70	1.81
55	记者捡起了铅笔	记者	作家	电台	宇宙
	The journalist picked up the pencil	journalist	writer	radio station	universe
	frequency	3.23	3.00	2.76	3.00
	relatedness		3.42	4.15	2.24
56	小孩错过了火车	小孩	大人	玩具	地球
	The kid missed the train	kid	adult	toy	earth
	frequency	3.59	3.44	3.11	3.36
	relatedness		5.25	4.42	2.12
57	厨房安装了空调	厨房	浴室	饭菜	飞船
	The kitchen was equipped with the air conditioner	kitchen	bathroom	meal	spaceship
	frequency	3.21	2.88	2.04	2.88
	relatedness		4.21	4.88	1.35
58	警察写下了地址	警察	保安	手铐	空气
	The policeman wrote down the address	policeman	security guard	handcuffs	air
	frequency	4.05	3.13	2.66	3.14
	relatedness		3.54	5.22	2.15
59	公主扔掉了木偶	公主	王子	城堡	地板
	The princess threw away the puppet	princess	prince	castle	floor
	frequency	3.09	3.03	2.84	2.99
	relatedness		4.17	5.15	2.90
60	坦克压折了树木	坦克	货车	炮弹	广告
	The tank burst into the woods	tank	truck	bomb	advertisement
	frequency	2.52	2.80	2.18	3.37
	relatedness		3.63	4.67	1.23
